# Identification of *Theileria* spp. and investigation of hematological profiles of their infections in goats in Hainan Island, China

**DOI:** 10.1051/parasite/2022013

**Published:** 2022-03-07

**Authors:** Lu Yang, Jin-Hua Wang, Archana Upadhyay, Jian-Guo Zhao, Liang-Yuan Huang, Cheng-Hong Liao, Qian Han

**Affiliations:** 1 Laboratory of Tropical Veterinary Medicine and Vector Biology, School of Life Sciences, Hainan University Haikou Hainan 570228 PR China; 2 One Health Institute, Hainan University Haikou Hainan 570228 PR China; 3 College of Animal Science and Technology, Hainan University Haikou Hainan 570228 PR China

**Keywords:** *Theileria*, Epidemiology, Hematological profile, Goat, Hainan

## Abstract

*Theileria* spp. are a group of parasites primarily transmitted by ticks and can pose a significant threat to domestic and wild animals globally. The main objective of this study was to understand the epidemiology of *Theileria* spp. in goats of Hainan Island/province, which is the only tropical region of China, and to study their hematological profiles in naturally infected goats. A total of 464 blood samples were collected from randomly selected local adult goats (*Capra hircus*, local domestic breed with black hair), from six cities and eight counties of Hainan, from November 2017 to October 2020. Blood smear microscopy of the sample and a nested polymerase chain reaction (nPCR) targeting the 18S rRNA gene combined with DNA sequencing were used to detect piroplasm infections in goats. Data analysis of the obtained sequences revealed that all the sequences were highly similar to the *Theileria luwenshuni* 18S rRNA gene sequence from the database. This result is consistent with the microscopic examination. In the hematological test, hematocrit, mean corpuscular volume, and mean corpuscular hemoglobin of the goats naturally infected with *T. luwenshuni* significantly increased, while mean corpuscular hemoglobin concentration and red blood cell distribution width (RDW) were significantly decreased. Results showed that *T. luwenshuni* could cause macrocytic, hypochromic anemia in goats. This study provides reliable and comprehensive information about the epidemiology of the parasite infections and hematological profile of the infected goats in Hainan, which encourages further investigations to develop practical control strategies for *Theileria* spp. infections in tropical areas.

## Abstract (fr)

Les *Theileria* spp. sont un groupe de parasites principalement transmis par les tiques qui peuvent constituer une menace importante pour les animaux domestiques et sauvages dans le monde. L’objectif principal de cette étude était de comprendre l’épidémiologie de *Theileria* spp. chez les chèvres de l’île/province de Hainan, qui est la seule région tropicale de Chine et étudier les profils hématologiques des chèvres naturellement infectées. 464 échantillons de sang ont été prélevés sur des chèvres adultes locales sélectionnées au hasard (*Capra hircus*, race domestique locale à poils noirs), dans 6 villes et 8 comtés de Hainan, de novembre 2017 à octobre 2020. L’étude microscopique du frottis sanguin de l’échantillon et la réaction en chaîne par polymérase nichée (nPCR) ciblant le gène de l’ARNr 18S combinée au séquençage de l’ADN ont été utilisées pour détecter les infections à piroplasmes chez les chèvres. L’analyse des séquences obtenues a révélé que toutes les séquences étaient très similaires à la séquence du gène de l’ARNr 18S de *T. luwenshuni* de la base de données. Le résultat est cohérent avec l’examen microscopique. Dans le test hématologique, l’hématocrite, le volume corpusculaire moyen et l’hémoglobine corpusculaire moyenne des chèvres naturellement infectées par *T. luwenshuni* ont augmenté de manière significative, tandis que la concentration moyenne d’hémoglobine corpusculaire et la largeur de distribution des globules rouges (RDW) ont été significativement diminuées. Les résultats ont montré que *T. luwenshuni* pouvait provoquer une anémie macrocytaire et une anémie hypochrome chez les chèvres. Cette étude fournit des informations fiables et complètes sur l’épidémiologie des infections parasitaires et le profil hématologique des chèvres infectées à Hainan, ce qui encourage des investigations supplémentaires pour développer des stratégies pratiques de contrôle des infections par *Theileria* spp. dans les zones tropicales.

## Introduction

*Theileria* spp. are tick-borne protozoan blood parasites of the Phylum Apicomplexa, Order Piroplasmida. Most of them possess a unique form of organelle that comprises a type of plastid called an apicoplast and an apical complex structure. *Theileria* spp. are mainly transmitted by ixodid ticks belonging to the genera *Amblyomma*, *Haemaphysalis*, *Hyalomma*, and *Rhipicephalus*. They infect a wide range of domestic and wild animals, especially tropical and subtropical ruminants [15]. Infestation can be a significant cause of economic losses because of severe disease outbreaks, high mortality rates, and reduced production.

*Theileria lestoquardi* causes ovine piroplasmosis in Northern Africa, Southern Europe, and the Middle East [3, 9]. In China, the prevalence of *Theileria* spp. has been reported from several provinces [8, 24]. At least nine species of *Theileria* have been reported in previous studies [1, 5, 6, 8, 23]. *Theileria lestoquardi*, *T. luwenshuni* and *T. uilenbergi* may cause serious clinical symptoms in sheep [8, 19, 23], while *T. ovis* and *T. separate* are less pathogenic or non-pathogenic [1]. Of the *Theileria* species present in China, *T. luwenshuni* and *T. uilenbergi* are the most prevalent in sheep and goats and are considered the most infective [12, 13, 24]. Hainan island/province is a tropical area in the south of China, where goats are common breeding ruminants. Minimal epidemiological information about *Theileria* spp. in goats from Hainan is available.

Goats infected with *Theileria* spp. display a wide range of clinical symptoms and signs, including fever, anorexia, weight loss, lymphadenopathy, respiratory signs (coughing, nasal discharge, dyspnea), anemia, icterus, and diarrhea [14]. Furthermore, similar symptoms have been reported in sheep infected with *T. luwenshuni* or *T. uilenbergi*. Hematological profiles may provide valuable information for the diagnosis, surveillance, and formulation of the prognosis of the disease in an individual [18]. Hence, the objective of this study was to investigate epidemiological information on *T. luwenshuni* and the changes in the hematological profiles related to *T. luwenshuni* infection in goats in Hainan.

## Material and methods

### Sample collection and DNA extraction

**Figure 1:**
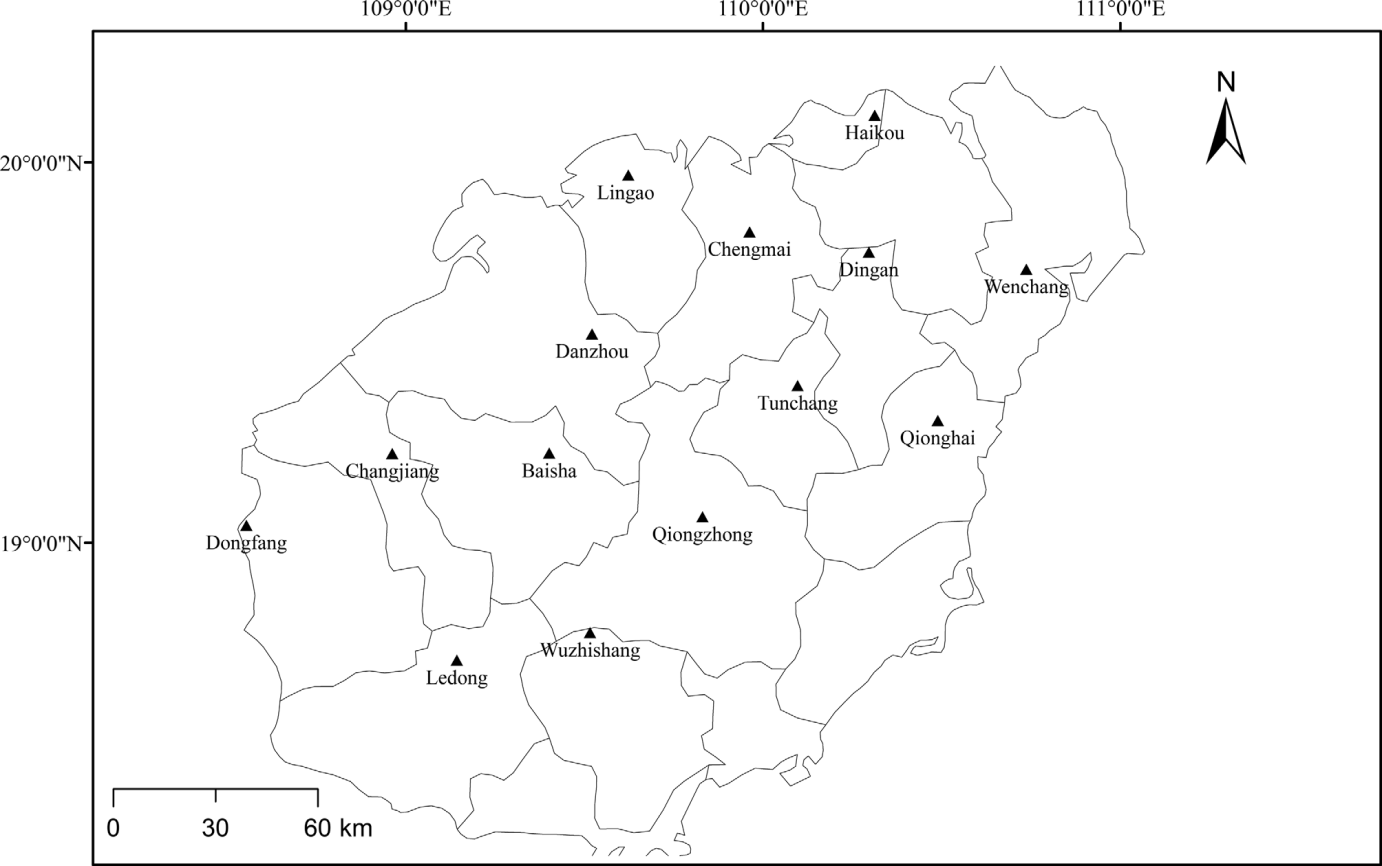
Map of Hainan showing locations where the samples were collected.

A total of 464 samples from black goats from six cities and eight counties were collected from selected sites across Hainan from November 2017 to October 2020 (Fig. 1). Blood samples were randomly collected from black goats, transferred into EDTA-coated vacuum tubes, and transported to the laboratory, maintaining cold conditions. According to the manufacturer’s instructions, genetic DNA was extracted using 100 μL of blood sample (Sangon Ezup Column blood genomic DNA extraction kit, China). The extracted DNA samples were stored at −20 °C until use.

### Blood smear microscopy

The collected blood of the black goats was used to prepare the blood smear, and Liu’s Stain (Solarbio, Beijing, China) was used, according to the manufacturer’s instructions. Then, thin blood smears were examined for the presence of intra-erythrocytic piroplasms of *Theileria* spp. under oil immersion (100× magnification) [16].

### Nested PCR amplification and DNA sequencing

Amplifications of the 18S rRNA gene were achieved by nPCR to check the presence of piroplasm infections in 464 samples. The first round of primers were universal eukaryotic primers for 18SrRNA genes [10]. The primers were T.lu-18S rRNA-F: 5′ – AACCTGGTTGATCCTGCCAGTAGT – 3′ and T.lu-18S rRNA-R: 5′ – GATCCTTCTGCAGGTTCACCTAC – 3′. The expected product size for the first round of PCR was approximately 1745 bp. Universal primers for *Babesia* spp. and *Theileria* spp. were used in the nested round of PCR [7]. The primers used for the nPCR were TBall (TBall-F: 5′ – GATAAC-CGTGCTAATTGTAGG – 3′; TBall-R: 5′ – ATCGTCTTCGATCCCCTA ACT – 3′). The expected amplicon size was approximately 808 bp. PCR amplifications were performed using 2 μL of DNA template, 25 μL of Green Taq Mix (2×) (Vazyme, Beijing, China), 0.5 μL of 10 μM forward and reverse primers of T.lu-18S rRNA. Double distilled water was added to make up the total reaction volume. PCR was performed using the following thermal cycling conditions: initial denaturation at 95 °C for 5 min; 40 cycles of 95 °C 30 s, 58 °C 30 s, 72 °C 90 s, finally extended at 72 °C for 10 min. Further, nested PCR was carried out using the second set of primers and 2 μL of product from the first round of PCR as the template DNA. Thermal cycling conditions for the second round or nested round of PCR were: initial denaturation step at 95 °C 5 min, 14 cycles at 95 °C for 30 s, 62 °C for 30 s subtracting 0.5 °C per cycle to 55 °C and 72 °C for 30 s, then 25 cycles at 95 °C or 20 s, 55 °C for 30 s and 72 °C for 30 s, with a final extension step at 72 °C for 10 min. PCR products were examined on 1% agarose gel stained with 0.1% GoldenView using a Quick-Load 5 kb DNA Ladder marker (TAKARA BIO, Inc. China), visualized under the Gel Doc XR^+^ Imaging system (Bio-Rad Laboratories, Inc.). All amplified PCR products were purified using a DNA gel purification kit (Sangon prep Kit) according to the manufacturer’s instructions and were sent to Sangon Biotech and Bio-engineering, Guangzhou, for DNA sequencing.

### Sequence analysis and phylogenetic analysis

All the obtained DNA sequences were analyzed using BLAST (http://blast.ncbi.nlm.nih.gov/Blast.cgi). All the sequences generated from this study were compared with the corresponding 18S rRNA reference sequences originating from BLAST analysis by Lasergene 8.0 software (http://DNASTAR.com) to verify the accuracy of the results. The obtained sequences of the 18S rRNA gene of piroplasm were also aligned with the reference sequences from GenBank using BioEdit software for Clustal W Multiple alignment algorithm (Number of bootstraps: 1000). A phylogenetic tree was constructed for phylogenetic tree analysis by the maximum likelihood method (Tamura-Nei model) using MEGA 7.0 software [22]. This result was confirmed using 1000 bootstrap replications.

### Hematological analysis

Fifty blood samples from the 464 samples were examined using a VetScan HM5 automatic blood cell analyzer (Abaxis, USA). Hematologic parameters were analyzed and calculated. Positive and negative samples were bifurcated into groups based on nPCR assay results. The negative group consisted of 35 healthy black goats and the positive group of 15 infected black goats. Statistical software SPSS 23.0 was used to conduct one-way ANOVA on various hematological indicators of the positive and negative groups to determine whether there were significant changes. We first used a homogeneity test of variances to infer the credibility of the data, and means were compared by Tukey’s and Duncan’s multiple range tests at a probability level ≤ 0.05. The reference values are provided by the Merck Veterinary Manual [21].

## Results

### Blood smear microscopy

**Figure 2:**
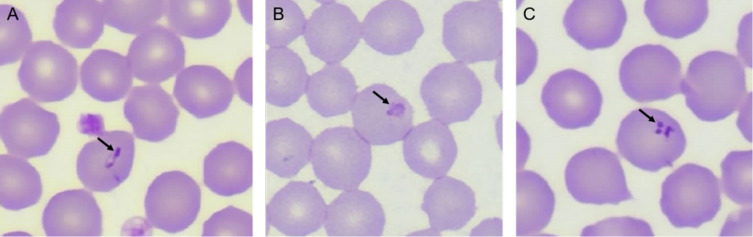
Peripheral blood smear examination showing the sporozoites of *T. luwenshuni* inside RBCs. A–C was magnified 2000×.

The microscopic examination of blood smear results showed that there were pear-seed-shaped, needle-shaped, rod-shaped, and cross-shaped parasites in red blood. The cytoplasm was light blue and the nucleus is dark purple (Fig. 2). This is consistent with the morphology reported in the literature. Therefore, the infestation was preliminarily diagnosed to be *Theileria* spp.

### PCR amplification

It was found that 161/464 DNA samples (34.7%) from black goats were positive for piroplasms. The obtained sequences showed > 99.9% nucleotide sequence identity to *T. luwenshuni* sequences deposited in NCBI GenBank by BLAST analysis. The results demonstrated that all piroplasm animals were also positive for *T. luwenshuni.* Among the 14 sampling sites, Chengmai (100%, 20/20), Dingan (62.2%, 56/90), and Wenchang (65.1%, 28/43) had a high prevalence of *Theileria* spp. infection, while Wuzhishan (0%, 0/21), Ledong (0%, 0/38), Changjiang (0%, 0/13), and Lingao (0%, 0/33) had no cases of infection (Table 1).

**Table 1:** Detection of *Theileria* spp.by the nPCR in goats from Hainan.

Cities/counties	No. of blood samples	No. of the positive	Prevalence of infections (%)
Baisha	29	6	20.7
Qiongzhong	9	3	33.3
Dongfang	23	6	26.1
Chengmai	20	20	100
Qionghai	29	8	27.6
Dingan	90	56	62.2
Wenchang	43	28	65.1
Danzhou	29	9	31.0
Haikou	48	13	27.1
Wuzhishang	21	0	0
Tunchang	39	12	30.8
Ledong	38	0	0
Lingao	33	0	0
Changjiang	13	0	0
Total	464	161	34.7

### Phylogenetic analysis

**Figure 3:**
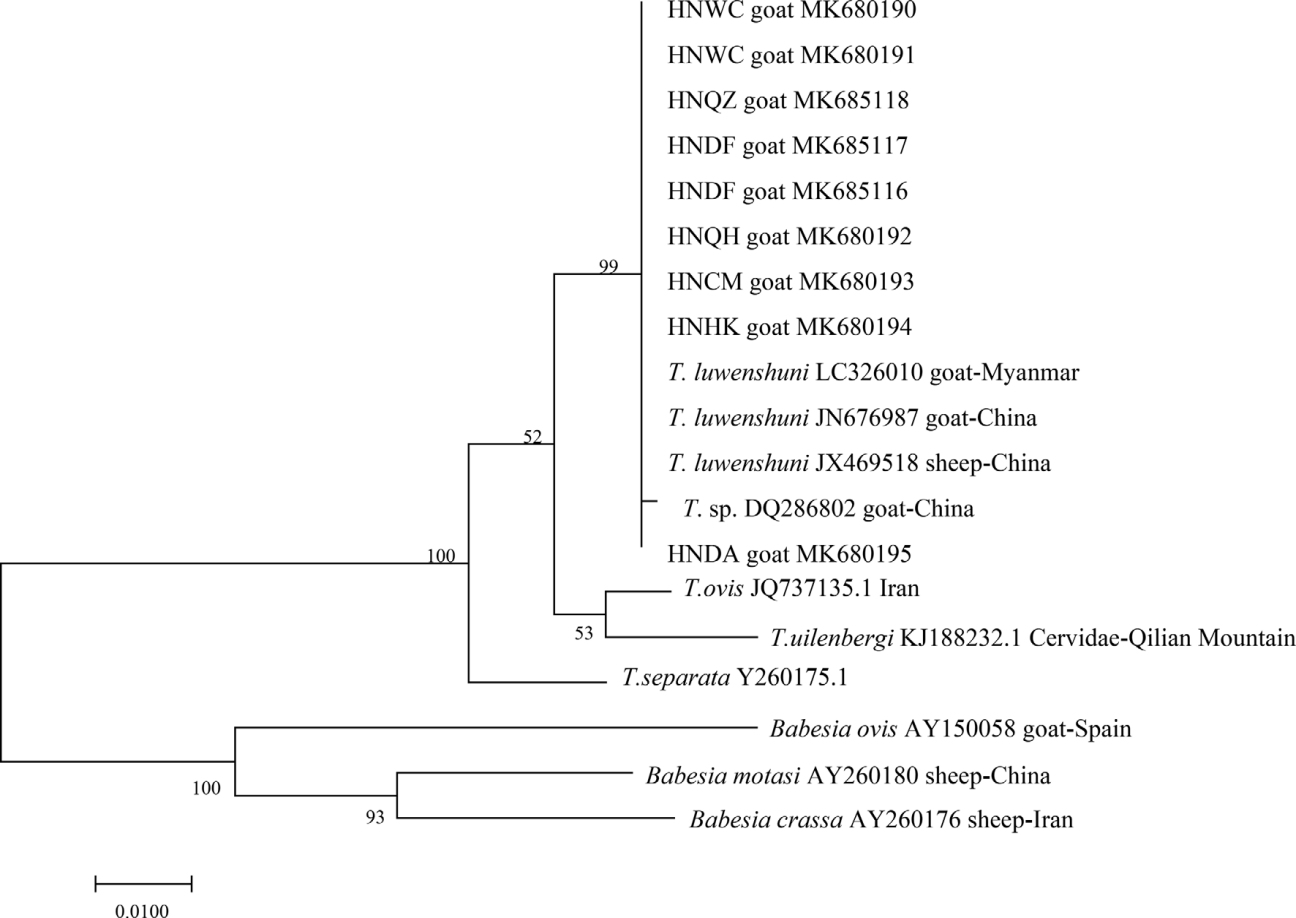
Phylogenetic tree of 18SrRNA of *T. luwenshuni* in black goats isolated from Hainan Island/province. The sequences obtained in the present study are indicated by “HN”. Bootstrap values are provided at the beginning of each branch.

A phylogenetic tree was constructed based on the sequences obtained in this study and sequences retrieved from the GenBank database (Fig. 3). In all, 19 sequences of the 18S rRNA gene of piroplasms in goats, including nine sequences of *T. luwenshuni* from the black goats (from Haikou, Dingan, Chengmei, Qionghai and Wenchang, Qiongzhong, Dongfang) obtained in this study, three *T. luwenshuni* gene sequences, and seven other sequences of piroplasms from the GenBank database were used for phylogenetic tree construction. The accession numbers are MK680190 – MK680195, MK685116 – MK685118. The phylogenetic analysis showed that all the sequences from Hainan black goats clustered in one clade with *T. luwenshuni* in China (JN676987) and Myanmar (LC326010) with 99% identity, and were different from other piroplasm sequences obtained from GenBank.

### Hematological analysis

To confirm whether *T. luwenshuni* infection induced alterations in the hematological parameters during infection, hematological parameters were compared between the negative and positive groups of *T. luwenshuni-*infected goats. The data were analyzed by statistical software SPSS 23.0, and the homogeneity of variance test proved that *T. luwenshuni* infection can affect the hematological parameters in the goats, but the differences were not significant (*p* > 0.05). Table 2 presents the hematological parameters of the goat blood samples from the negative and positive groups. The mean values had significant differences in hematocrit (HCT), mean cell volume (MCV), mean cell hemoglobin (MCH), mean cell hemoglobin concentration (MCHC), and red cell distribution width (RDW) between the negative and positive groups. Among them, HCT, MCV, and MCH were significantly increased (*p* < 0.01), while MCHC and RDW were significantly decreased (*p* < 0.01).

**Table 2:** The averages (SD) of hematological parameters of *T. luwenshuni* positive and negative samples in goats.

	Negative (*n* = 35)	Positive (*n* = 15)	Reference values	*P*-value
WBC 10^9^/L	17.95	20.18	4.0–13.0	0.170
RBC 10^12^/L	16.10	15.82	8.0–18.0	0.375
NEU %	59.69	58.35	30–40	0.645
HGB g/L	9.12	9.68	8–12	0.178
HCT %	24.60	27.31	25–38	0.008
MCV fL	15.26	17.13	16–25	0.000
MCH pg	5.69	6.13	5.2–8.0	0.000
MCHC g/dL	37.02	35.43	30–36	0.001
RDW %	34.97	32.71	30–45	0.002

## Discussion

In the present study, we investigated the epidemiology of *Theileria* spp. in goats from Hainan. We also explored the association between *T. luwenshuni* infection and hematological parameters. To our knowledge, this is the first report on epidemiological investigations related to piroplasm infections in goats from Hainan.

Goat piroplasmosis is a tick-borne disease caused by apicomplexan parasites, including *Theileria* and *Babesia*. In this study, the parasite bodies of *Theileria* spp. were found by blood smear microscopy, which was consistent with the morphology of *Theileria* spp. described in the literature [11, 25], so *Theileria* spp. was preliminarily diagnosed as the infecting species. All samples were then tested using nPCR. A sample of 464 goats from 14 different sampling sites in Hainan were included in the study. In some areas, the infection rate of piroplasm in goats was as high as 100%. The average prevalence of the piroplasm was 34.7%. Sequencing results showed that *T. luwenshuni* was the most prevalent parasite found in goats in Hainan. The results were consistent with those obtained by blood smear microscopy. In addition, this study found that different feeding methods were correlated with the disease’s infection rate. The infection rate in grazing goats is significantly higher than that of house-fed goats. Presumably, this is because house-fed animals are less likely to be bitten by ticks. The finding of only *T. luwenshuni* infection in goats in Hainan suggested it may be the dominant piroplasm infecting goats in this island. Piroplasm infection in goats should be related to the number and distribution of the piroplasm vectors. There are around 5 genera and 12 species of ticks found in Hainan, and *Haemaphysalis longicornis* has been reported in ruminants from Qiongzhong and Wanning, Hainan. *Haemaphysalis longicornis* is one of the main vectors of *Theileria* spp., which is mainly distributed across the northeast and the southeast of China. *Rhipicephalus sanguineus* and *Boophilus microplus* have also been found abundantly in Hainan (unpublished data). Therefore, tick-borne diseases can easily be transmitted and can thrive well in tropical climates where the climate is favorable for tick survival.

Phylogenetic analysis of the sequences revealed the similarity of the obtained sequences from Hainan to the corresponding sequences of *T. luwenshuni* from other regions of China (accession numbers: GenBank sequence JN676987 and JX469518) and the Myanmar isolate (GenBank sequence: LC326010), which further proves that the strain obtained from Hainan was *T. luwenshuni* [4].

The hematological index of animals is an important indicator reflecting their physiological function and an essential basis for the clinical diagnosis of diseases. *Theileria luwenshuni* is widely distributed in China and highly pathogenic in goats [4, 17]. In this study, hematologic tests were performed on both naturally infected and uninfected goats. It was found that MCV and MCH increased significantly, while MCHC decreased significantly. Therefore, the condition is classified as macrocytic, hypochromic anemia. This is consistent with the presentation of tick-borne blood parasitic disease reported in other literature [2]. However, the significant increase of MCH in this study is inconsistent with the substantial decrease in MCH reported by Shruthi [20], which may be due to that the different infecting *Theileria* species. However, this needs to be investigated in subsequent studies. In this study, there was no significant difference in red blood cell (RBC) and hemoglobin (HGB) levels, which was different from the significant decrease in RBC and HGB levels reported in the literature [14]. This is speculated to be due to the fact that the goats infected with *T. luwenshuni* in the investigation were not in the prometaphase of acute infection, but in a carrying state in the later stage of infection.

To our knowledge, this is the first molecular and epidemiological report of piroplasm infection in the goats from Hainan. Hematological tests showed that the infected goats may have macrocytic, hypochromic anemia. This investigation and data are of great significance and importance for preventing, controlling, and managing piroplasmosis in ruminants in Hainan, which encourages more detailed studies in the future.

## References

[1] Aktaş M, Altay K, Dumanli N. 2005. Survey of *Theileria* parasites of sheep in eastern Turkey using polymerase chain reaction. Small Ruminant Research, 60(3), 289–293.

[2] Ali Shah S, Khan M, Rahman H. 2017. Epidemiological and hematological investigations of tick-borne diseases in small ruminants in Peshawar and Khyber Agency, Pakistan. Journal of Advances in Parasitology, 4, 15–22.

[3] Aydin MF, Aktas M, Dumanli N. 2013. Molecular identification of *Theileria* and *Babesia* in sheep and goats in the Black Sea Region in Turkey. Parasitology Research, 112(8), 2817–2824. 23689604 10.1007/s00436-013-3452-x

[4] Bawm S, Kakisaka K, Thu MJ, Chel HM, Oo YMN, Soe NC, Win SY, Htun LL, Win MM, Suzuki H, Nakao R, Katakura K. 2018. First molecular detection of *Theileria luwenshuni* from goats in Myanmar. Parasitol Research, 117(10), 3361–3364. 10.1007/s00436-018-6073-6 30187170

[5] Cao S, Zhang S, Jia L, Xue S, Yu L, Kamyingkird K, Moumouni PF, Moussa AA, Zhou M, Zhang Y, Terkawi MA, Masatani T, Nishikawa Y, Xuan X. 2013. Molecular detection of *Theileria* species in sheep from northern China. Journal of Veterinary Medical Science, 75(9), 1227–1230. 10.1292/jvms.13-0028 23594412

[6] Ge Y, Pan WQ, Yin H. 2012. Prevalence of *Theileria* infections in goats and sheep in southeastern China. Veterinary Parasitology, 186(3–4), 466–469. 22178410 10.1016/j.vetpar.2011.11.066

[7] Guan G, Moreau E, Liu J, Hao X, Ma M, Luo J, Chauvin A, Yin H. 2010. *Babesia* sp. BQ1 (Lintan): molecular evidence of experimental transmission to sheep by *Haemaphysalis qinghaiensis* and *Haemaphysalis longicornis*. Parasitology International, 59(2), 265–267. 20026243 10.1016/j.parint.2009.12.002

[8] Guo S, Yuan Z, Wu G, Wang W, Ma D, Du H. 2002. Epidemiology of ovine theileriosis in Ganan region, Gansu Province, China. Parasitology Research, 88(13 Suppl 1), S36–S37. 12051605 10.1007/s00436-001-0568-1

[9] Iqbal F, Khattak R, Ozubek S, Khattak M, Rasul A, Aktas M. 2013. Application of the reverse line blot assay for the molecular detection of *Theileria* and *Babesia* sp. in sheep and goat blood samples from Pakistan. Iranian Journal of Parasitology, 8(2), 289–295. 23914243 PMC3724155

[10] Koid A, Mraz A. 2012. Comparative analysis of eukaryotic marine microbial assemblages from 18S rRNA gene and gene transcript clone libraries by using different methods of extraction. Applied & Environmental Microbiology, 78(11), 3958. 22447590 10.1128/AEM.06941-11 PMC3346388

[11] Li Y, Liu J, Liu Z, Yang J, Li Y, Li Q, Qin G, Chen Z, Guan G, Luo J, Yin H. 2015. Report of *Theileria luwenshuni* and *Theileria* sp. RSR from cervids in Gansu, China. Parasitology Research, 114(5), 2023–2029. 25820647 10.1007/s00436-015-4439-6

[12] Li Y, Zhang X, Liu Z, Chen Z, Yang J, He H, Guan G, Liu A, Ren Q, Niu Q. 2014. An epidemiological survey of *Theileria* infections in small ruminants in central China. Veterinary Parasitology, 200(1–2), 198–202. 24365241 10.1016/j.vetpar.2013.07.023

[13] Liu Z, Hou J, Bakheit MA, Salih DA, Luo J, Yin H, Ahmed JS, Seitzer U. 2008. Development of loop-mediated isothermal amplification (LAMP) assay for rapid diagnosis of ovine theileriosis in China. Parasitology Research, 103(6), 1407–1412. 18751728 10.1007/s00436-008-1149-3

[14] Mahmoud M, Al-Dhalimy A, Al-Dujaily A. 2019. Study of hematological and biochemical changes in sheep and goats infected with theileriosis AT-Najaf province, Iraq. Biochemical and Cellular Archives, 19(1), 1863–1867.

[15] Mehlhorn H, Shein E. 1984. The piroplasms: life cycle and sexual stages. Advances in Parasitology, 23, 37–103. 6442536 10.1016/s0065-308x(08)60285-7

[16] Ozubek S, Aktas M. 2017. Molecular and parasitological survey of ovine piroplasmosis, including the first report of *Theileria annulata* (Apicomplexa: Theileridae) in sheep and goats from Turkey. Journal of Medical Entomology, 54(1), 212–220. 28082649 10.1093/jme/tjw134

[17] Phipps LP, Hernandez-Triana LM, Goharriz H, Welchman D, Johnson N. 2016. Detection of *Theileria luwenshuni* in sheep from Great Britain. Parasites & Vectors, 9, 203. 27075661 10.1186/s13071-016-1486-5 PMC4831081

[18] Roland L, Drillich M, Iwersen M. 2014. Hematology as a diagnostic tool in bovine medicine. Journal of Veterinary Diagnostic Investigation, 26(5), 592–598. 25121728 10.1177/1040638714546490

[19] Seitzer U, Bakheit MA, Salih DE, Ali A, Haller D, Yin H, Schnittger L, Ahmed J. 2007. From molecule to diagnostic tool: *Theileria annulata* surface protein TaSP. Parasitology Research, 101(Suppl 2), S217–S223. 17823831 10.1007/s00436-007-0685-6

[20] Shruthi R, Thimmareddy PM, Mamatha GS, Chandranaik BM, Puttalakshmamma GC. 2017. Studies on theileriosis in goats from Karnataka, South India. Journal of Parasitic Diseases, 41(4), 1082–1085. 29114145 10.1007/s12639-017-0937-z PMC5660037

[21] Soul W, Mupangwa J, Muchenje V, Mpendulo TC. 2019. Biochemical indices and hematological parameters of goats fed *Lablab purpureus* and *Vigna unguiculata* as supplements to a *Chloris gayana* basal diet. Veterinary and Animal Science, 8, 100073. 32734090 10.1016/j.vas.2019.100073 PMC7386721

[22] Sudhir K, Glen S, Koichiro T. 2016. MEGA7: molecular evolutionary genetics analysis version 7.0 for bigger datasets. Molecular Biology & Evolution, 33(7), 1870. 27004904 10.1093/molbev/msw054 PMC8210823

[23] Tian ZC, Liu GY, Yin H, Luo JX, Guan GQ, Luo J, Xie JR, Zheng JF, Yuan XS, Wang FF, Shen H, Tian MY. 2013. Discrimination between ovine *Babesia* and *Theileria* species in China based on the ribosomal protein S8 (RPS8) gene. Veterinary Parasitology, 197(1–2), 354–359. 23747103 10.1016/j.vetpar.2013.04.033

[24] Yin H, Liu Z, Guan G, Liu A, Ma M, Ren Q, Luo J. 2008. Detection and differentiation of *Theileria luwenshuni* and *T. uilenbergi* infection in small ruminants by PCR. Transboundary and Emerging Diseases, 55(5–6), 233–237. 18666967 10.1111/j.1865-1682.2008.01031.x

[25] Zhang X, Liu Z, Yang J, Chen Z, Guan G, Ren Q, Liu A, Luo J, Yin H, Li Y. 2014. Multiplex PCR for diagnosis of *Theileria uilenbergi*, *Theileria luwenshuni*, and *Theileria ovis* in small ruminants. Parasitology Research, 113(2), 527–531. 24241125 10.1007/s00436-013-3684-9

